# Targeted sequencing of linkage region in Dominican families implicates *PRIMA1* and the *SPATA7-PTPN21-ZC3H14-EML5-TTC8* locus in carotid-intima media thickness and atherosclerotic events

**DOI:** 10.1038/s41598-019-48186-1

**Published:** 2019-08-12

**Authors:** Liyong Wang, Nicole Dueker, Ashley Beecham, Susan H. Blanton, Ralph L. Sacco, Tatjana Rundek

**Affiliations:** 1 John P Hussman Institute for Human Genomics, Miller School of Medicine, University of Miami, Miami, FL USA; 2 John T. McDonald Department of Human Genetics, Miller School of Medicine, University of Miami, Miami, FL USA; 3 Department of Neurology, Public Health Sciences, Miami, FL, USA; 4 Evelyn F. McKnight Brain Institute, Miller School of Medicine, University of Miami, Miami, FL USA

**Keywords:** Quantitative trait loci, Next-generation sequencing

## Abstract

Carotid intima-media thickness (cIMT) is a subclinical marker for atherosclerosis. Previously, we reported a quantitative trait locus (QTL) for total cIMT on chromosome 14q and identified *PRiMA1*, *FOXN3* and *CCDC88C* as candidate genes using a common variants (CVs)-based approach. Herein, we further evaluated the genetic contribution of the QTL to cIMT by resequencing. We sequenced all exons within the QTL and genomic regions of *PRiMA1*, *FOXN3* and *CCDC88C* in Dominican families with evidence for linkage to the QTL. Unrelated Dominicans from the Northern Manhattan Study (NOMAS) were used for validation. Single-variant-based and gene-based analyses were performed for CVs and rare variants (RVs). The strongest evidence for association with CVs was found in *PRiMA1* (p = 8.2 × 10^−5^ in families, p = 0.01 in NOMAS at rs12587586), and in the five-gene cluster *SPATA7-PTPN21-ZC3H14*-*EML5*-*TTC8* locus (p = 1.3 × 10^−4^ in families, p = 0.01 in NOMAS at rs2274736). No evidence for association with RVs was found in *PRiMA1*. The top marker from previous study in *PRiMA1 (*rs7152362) was associated with fewer atherosclerotic events (OR = 0.67; p = 0.02 in NOMAS) and smaller cIMT (β = −0.58, p = 2.8 × 10^−4^ in Family). Within the five-gene cluster, evidence for association was found for exonic RVs (p = 0.02 in families, p = 0.28 in NOMAS), which was enriched among RVs with higher functional potentials (p = 0.05 in NOMAS for RVs in the top functional tertile). In summary, targeted resequencing provided validation and novel insights into the genetic architecture of cIMT, suggesting stronger effects for RVs with higher functional potentials. Furthermore, our data support the clinical relevance of CVs associated with subclinical atherosclerosis.

## Introduction

Carotid intima-media thickness (cIMT) assessed by high-resolution ultrasound is a noninvasive, inexpensive and highly reproducible measure of subclinical atherosclerosis. Atherosclerosis continues to be the leading cause of overt vascular diseases. cIMT is a useful precursor phenotype of vascular diseases, which are heterogeneous and extremely complex conditions with both genetic and environmental contributions. Evaluation of cIMT may reduce phenotypic heterogeneity and increase statistical power in genetic studies of vascular diseases. Heritability studies suggest a strong genetic contribution to cIMT, with estimated heritability ranging from 0.3 to 0.6 in various populations^1–4^. Characterizing the genetic architecture of cIMT might provide insights into the pathogenesis of vascular diseases and identify individuals at higher risk of vascular events in preclinical stage.

Large scale genome-wide association studies (GWAS) have been carried out for cIMT with a focus on common genetic variants. *ZHX2*, *APOC1*, *PINX*, *SLC17A4*, *BCAR1-CFDP1-TMEM170A* locus have been associated with common cIMT in European cohorts^5,6^. The first GWAS for cIMT in a non-European population was reported in 772 Mexican Americans from the San Antonio Family Heart Study but no genome-wide significant signal was found^7^. In addition to GWAS, a few recent studies have included rare genetic variants in the study of cIMT. The CHARGE consortium re-sequenced candidate genes identified in the GWAS meta-analysis. Neither single variant analysis for common variant (CV) nor gene-based rare variant (RV) analysis remained significant after adjusting for multiple testing^8^. The Paris Prospective Study 3 (PPS3) used exome chips to examine associations between genetic variants and several carotid phenotypes, including cIMT. Although the genetic variants included in the exome array seemed to account for a significant portion of cIMT variation (~10%), no single variant or gene was significantly associated with cIMT^9^.

We have mapped a quantitative trait locus (QTL) for total cIMT (a composite measure of cIMT of the near and the fall wall of IMT from all carotid sites) to chromosome 14q using 100 extended Dominican Republic (DR) families in a genome-wide linkage study^10^. We also identified *PRiMA1*, *FOXN3* and *CCDC88C* as candidate genes in a peak-wide association study using tagSNPs (single nucleotide polymorphisms) to survey common variants (CVs) within the QTL^11^. In this study, we carried out a targeted re-sequencing study of the QTL to comprehensively evaluate the contribution of CVs and RVs to cIMT. DR families with evidence for linkage to the QTL from the original linkage analysis were sequenced. We also included an independent cohort of Dominicans from the Northern Manhattan Study (NOMAS) to facilitate filtering for the most promising findings.

## Materials and Methods

### Sample sets

Two sample sets were constructed using participants from our NIH-funded Family Study of Stroke Risk and Carotid Atherosclerosis (100 families, 1360 participants) and the population-based Northern Manhattan Study (NOMAS) consisting of 3,298 community dwelling participants^10,12,13^. The probands for the Family Study were selected from NOMAS Caribbean Hispanic members at high risk for stroke and cardiovascular disease^12^. Families were enrolled if the proband was able to provide a family history, obtain the family members’ permission for the research staff to contact them, and had at least three first-degree relatives able to participate.

The discovery sample set includes seven Dominican Republican (DR) families with a family-specific LOD score >0.1 at the chromosome 14 QTL for total cIMT^10^. The validation sample set includes unrelated Dominicans from NOMAS with available cIMT and genotype data (N = 561). Demographic, socioeconomic and risk factor data were collected through direct interview based on the NOMAS instruments.

### Ethics statement

The study was designed and implemented following the principles outlined in the Nuremberg Code and Belmont Report. The research protocol was approved by the Institutional Review Boards (IRB) of Columbia University, University of Miami, and the National Bioethics Committee, and the Independent Ethics Committee of Instituto Oncologico Regional del Cibao in the DR. All subjects provided written informed consent.

### Carotid measurements

All family members and NOMAS subjects had high resolution B-mode ultrasound assessments of carotid IMT as detailed previously^14^. The cIMT protocols yield measurements of the distance between lumen-intima and media-adventitia ultrasound echoes, from which the cIMT and arterial diameter are derived for the 3 carotid segments. cIMT measurements were performed outside the areas of plaque as recommended by an international consensus^15^. cIMT was measured using an automated computerized edge tracking software M’Ath (Intelligence in Medical Technologies, Inc., Paris, France) from the recorded ultrasound clips^16^. Total cIMT is calculated as a composite measure of the means of the near and the far wall IMT of all carotid sites (the common carotid artery, the internal carotid artery and the bifurcation) from both sides of the neck. Our cIMT measurements have excellent consistency; inter-reader reliability between 2 readers was demonstrated with a mean absolute difference in cIMT of 0.11 ± 0.09 mm, variation coefficient 5.5%, correlation coefficient 0.87, and the percent error 6.7%; intra-reader mean absolute cIMT difference was 0.07 ± 0.04 mm, variation coefficient 5.4%, correlation coefficient 0.94, and the percent error 5.6%^16^.

### Next generation sequencing (NGS) and genotyping

All sequencing and genotyping were carried out at Center for Genome Technology at the John P Hussman Institute for Human Genomics (HIHG) at University of Miami. Exons of genes within the 1 LOD-unit down intervals (77.7 Mb~95.0 Mb) of the chr 14 QTL, as well as introns, exons and 5 Kb flanking regions of *PRiMA1*, *FOXN3* and *CCDC88C* were sequenced in the discovery sample set. Targeted regions were captured with a customized Agilent SureSelect Enrichment kit. DNA libraries with 24-sample barcoding were sequenced on Illumina HiSeq2000 with pair-end sequencing at 100 base pair reading length. On average, each DNA sample generated about 15 million raw reads. The raw sequencing reads were processed via the bioinformatics pipeline at the Center for Genetic Technology at HIHG as described before^17^. Briefly, the raw sequencing reads were aligned to the human reference sequence hg19 with the Burrows-Wheeler Aligner (BWA)^18^ and variant calling was done with the Genome Analysis ToolKit (GATK)^18^. At a depth of 8, 97% of the target sequences were covered. Variants with VQSLOD < −4 were removed from analysis. Within each individual sample, variants with a depth < 4 or Phred-Like (PL) score <100 were set as missing. Variants with call rate <75% were also removed from further analysis. Concordance between the sequencing data and genotypes from the previous peak-wide association study were assessed for each sample. No samples had low concordance (<95%). Pedigree structure was confirmed using the Graphical Relationship Representation software. Mendelian error checking was performed and Mendelian errors were set to missing for all the variants called using PLATO^19^.

DNA from the replication NOMAS cohort was genotyped using the Genome-Wide Human SNP Array 6.0 chip (AffyMetrix) and HumanCoreExome-12v1.1 with custom content (Illumina). The Affymetrix 6.0 genotyping data were used to impute up to 38 million SNPs using IMPUTE2 with all ancestries in the 1000 genomes phase 1, version 3 reference panel^20^. Custom content included exonic single nucleotide variants (SNVs) selected from sequencing data obtained in the discovery sample set. Variants were selected if they: (1) were not included on the Illumina HumanCoreExome-12v1.1 Beadchip; (2) passed sequencing quality control in at least three individuals; (3) could not be effectively imputed using GWAS markers (imputation quality score < 0.8); (4) had an Illumina Infinium® design score > = 0.6. Genotype calling was performed using Illumina’s GenTrain version 1.0 clustering algorithm in GenomeStudio version 2011.1 and a GenCall cutoff score of 0.15 was used. Samples with call rates below 95%, relatedness or sex discrepancies, and outliers beyond 6 SD from the mean based on Eigenstrat analysis were removed from further analysis using PLINK 1.07^21^.

Variants were annotated for potential functions and allele frequency using Bravo (https://Bravo.sph.umich.edu), ANNOVAR^22^, RegulomeDB^23^, Combined Annotation-Dependent Depletion (CADD) score^24^, and HaploReg^25^. Variants were claimed “Novel” if they were not found in any publicly available databases.

### Statistical analysis

Total cIMT was natural log transformed to ensure a normal distribution as in previous analyses^10,11^. The covariates were identified using a polygenic screen. For the Family sample set, age, age^2^, sex, pack years of smoking, and waist-hip ratio were significant in the polygenic screen and were included as covariates. Family-based association tests (see below) were used for the Family dataset, which takes into account the linkage while testing for association. For the NOMAS sample set, age, age^2^, sex, diabetes, and the first principal component (PC1) estimated by the Eigenstrat were significant in the polygenic screen and were included as covariates. For association with atherosclerotic events in NOMAS, age, sex, PC1, hypertension, diabetes, BMI, pack years of smoking and dyslipidemia were included as covariates. An atherosclerotic event was defined as having a myocardial infarction, ischemic stroke or vascular death. For the Family Study, CVs and RVs were defined based on estimated frequencies from the population-based cohort of 591 Dominicans from NOMAS using Affymetrix 6.0 genotyping data. Most GWAS and our own previous studies focused on CVs have used an allele frequency cutoff of 5% for the variants to be included in the analysis. To be consistent with and complementary to previous studies, variants were defined as CVs if they had a minor allele frequency (MAF) ≥ 5% in NOMAS Dominicans and classified as RVs if they had MAF < 5% or could not be imputed efficiently (INFO ≤ 0.4) in NOMAS Dominicans, an indication that the variants are rare. All the variants used in the association analysis are either genotyped by NGS (for the Family Study) or genotyping chips (for NOMAS), including customized exome chip. For CVs, single variants were evaluated for association with total cIMT using the Quantitative Transmission-Disequilibrium test (QTDT) implemented in SOLAR^26^ for the Family sample set and the linear regression with an additive genetic model implemented in PLINK^21^ for the NOMAS sample set, as reported before^11^. The linkage disequilibrium (LD) between the CVs was estimated based on sequencing data from the family study using SOLAR, which is very similar to the LD estimates based on genotype data from Phase 3 of 1000 Genomes Project using the Admixed American (AMR) population^27^. Gene-based association analyses were performed to collapse all exonic RVs within each gene to improve statistical power. To infer if RVs with functional potential are more likely to contribute to the genetic associations, additional analyses were performed using different filtering algorithms based on the functional annotation of exonic RVs. Analyses were restricted to genes with ≥2 polymorphic variants. Family SNP-set (Sequence) Kernel Association Test (Fam-SKAT) and the SKAT-O were used for the family samples and NOMAS samples, respectively^28^.

In order to reduce false positive findings as a result of multiple testing, we deployed the two-stage design, i.e. using an independent replication dataset to validate suggestive findings in the discovery dataset and to minimize false positive rate. We used this approach instead of applying Bonferroni corrected P values because the Bonferroni correction is known for being overly conservative.

## Results

### Overview of single nucleotide variants (SNVs) identified by re-sequencing

We sequenced 116 individuals from seven DR families with a family-specific LOD score >0.1 at the chromosome 14 QTL (Table 1). The family size ranged from 11 to 25, with a median size of 15. In total, 6300 bi-allelic SNVs were observed in this sample set; 3869 were RVs and 2431 were CVs (Table 2). Of all the variants, 2.3% (89 variants) were novel (i.e., not found in public databases). All the novel variants were RVs and were more prevalent in the non-exonic regions and UTRs (N = 79), compared to the open-reading frame region (N = 10).

**Table 1:** Characteristics of Families Sequenced.

Family ID	Individuals per Family	Number of 1^st^ degree relatives	Family-Specific LOD	Total cIMT Residual*			
				Mean	SD	Min	Max
5987	14	2	0.70	0.92	1.03	−0.83	2.37
5623	15	3	0.55	−0.47	0.76	−1.65	0.96
3561	18	5	0.51	−0.06	0.74	−1.01	1.61
3630	13	6	0.24	−0.02	0.73	−1.38	1.74
803	11	1	0.20	−0.10	0.76	−1.56	1.63
5279	20	10	0.19	0.89	0.77	−0.70	2.26
5275	25	2	0.13	−0.10	0.62	−1.51	1.40

*Total cIMT residual was computed after adjusting for the significant risk factors (age, age^2^, sex, pack years of smoking, and waist-hip-ratio) using SAS.

**Table 2:** Single nucleotide variants identified by re-sequencing chr14 QTL in Dominicans.

	Non-exonic	UTRs	Synonymous	NonSynonymous	Total
Rare Variant	3182 (61)*	411 (18)	132 (4)	144 (6)	3869
Common Variant	2029	240	86	76	2431
Total	5211	768	246	256	6300

*The number in parenthesis is the number of variants that are novel. Targeted re-sequencing of chr14 QTL was done in the Family study. Variants were categorized into different allele frequency categories based on imputation data generated from NOMAS. Variants with an estimated minor allele frequency greater than or equal to 5% in the NOMAS cohort are classified by common variant. Variants with an estimated minor allele frequency less than 5% or not imputed efficiently (INFO ≤ 0.4) in the NOMAS cohort are classified as rare variants.

### Common variant analysis

**Figure 1:**
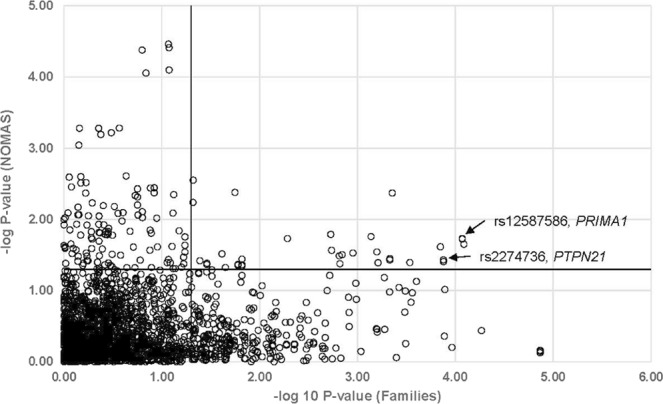
Single Variant Analysis of Common Variants in Families and NOMAS. Each circle represents the p-values of a single variant analysis of common variants (CV, MAF ≥ 0.05) in Families (X-axis) and NOMAS (Y-axis) datasets. The two solid lines depict p = 0.05 in each sample set, respectively. CVs that are significant in both datasets are located in the upper right section of the plot. The strongest association was found with rs12587586 (p = 8.2 × 10^−5^ in Families, p = 0.01 in NOMAS) in *PRiMA1*. The second strongest association was found with a missense SNP rs2274736 (p = 1.3 × 10^−4^ in Families and p = 0.01 in NOMAS) in *PTPN21*.

**Figure 2:**
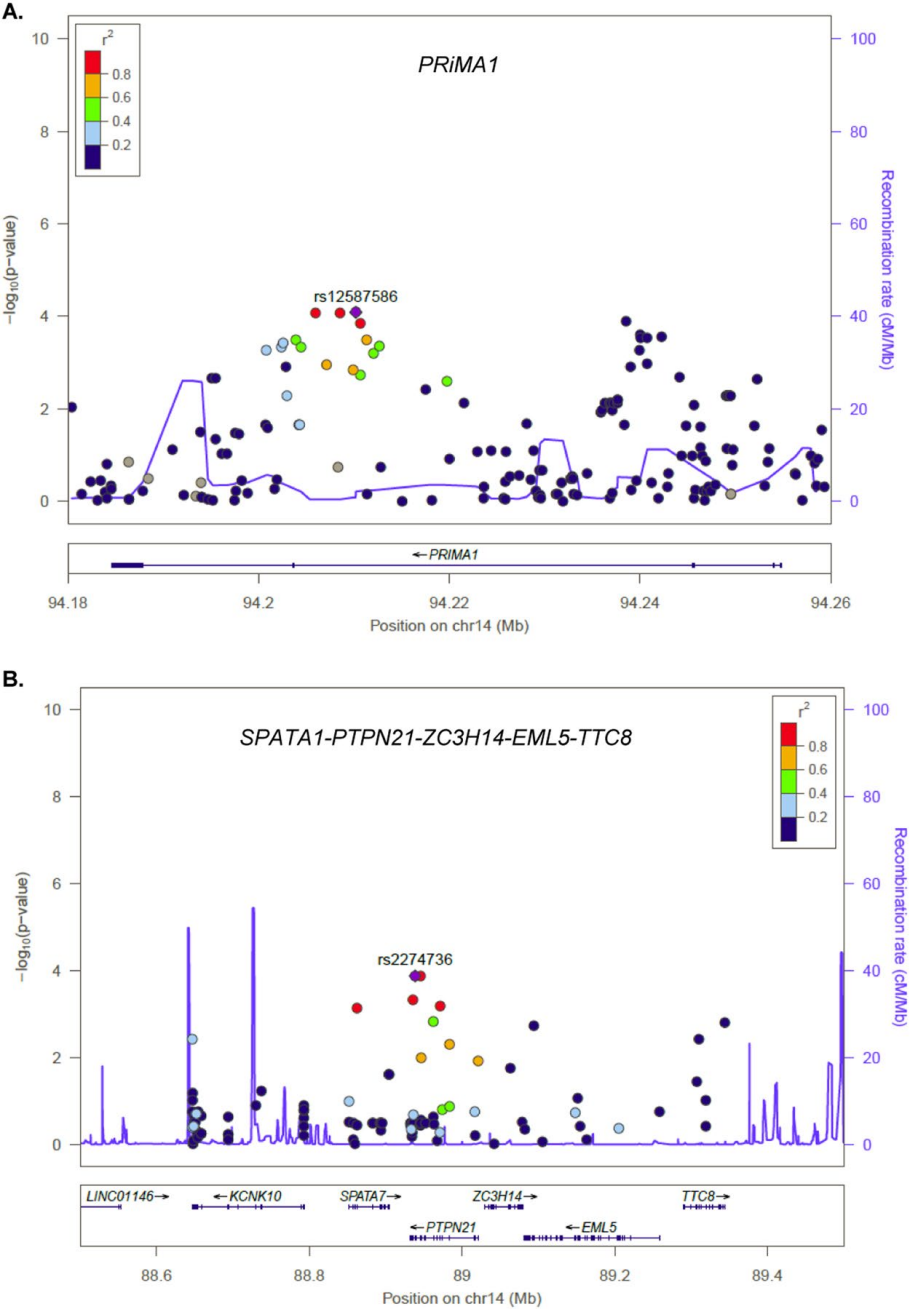
Regional Plots of *PRiMA1* and *PTPN21*. The X-axis displays the base pair location of each CV on chromosome 14; Y axis displays the –Log_10_ (p-values) of QTDT for association with total cIMT in the Families. The index CV is displayed as a purple diamond. The color of the circles indicates LD estimated in the families between each CV and the index CV. The solid purple line displays the estimated recombination rate. In *PRiMA1* (panel A.), the index CV is rs12587586, the top marker identified in the current study. The most significant marker in the previous study (rs7152362) is not in LD (r^2^ = 0.02; D′ = 0.31) with rs12587586. In *PTPN21*, the index CV is rs2274736, the most significant CV in the region. It is located within a region with extended LD containing five genes, i.e. *SPATA7*, *PTPN21*, *ZC3H14*, *EML5*, and *TTC8*.

Within the linkage region, 35 CVs were associated with cIMT in both the Family and NOMAS datasets (p ≤ 0.05 in both datasets) (Fig. 1). Among them, the strongest association was found to rs12587586 (p = 8.2 × 10^−5^ in Family, p = 0.01 in NOMAS) in the *PRiMA1* gene, which was the most significant gene in the previous study using tagSNPs^11^. Importantly, this marker was not in LD (r^2^ = 0.002; D’ = 0.1) with the top marker, rs7152362, reported in the previous study (Fig. 2a). Conditional analyses revealed that the two SNPs are independent of each other and both of them remain associated with cIMT after adjusting for another one (adjusted P = 0.0007 and 0.003 for rs12587586 and rs7152362, respectively). However, the effect size of rs12587586 is in the opposite direction in the discovery family data set and the validation NOMAS data set (β = −0.83 and 0.01, respectively), while the effect size of rs7152362 is in the same direction in the discovery family data set and the validation NOMAS data set (β = −0.58 and −0.01, respectively).

Both rs12587586 (MAF = 0.22) and rs7152362 (MAF = 0.41) are located in the 3^rd^ intron of *PRiMA1*. SNP rs12587586 is located within an active enhancer in brain and heart tissue with a RegulomeDB score of 5^23,25^. Among 10 proxy SNPs (pairwise r^2^ ≥ 0.7) of rs12587586, rs11848682 has the highest regulatory potential with a RegulomeDB score of 4^29^. It is located within enhancers in multiple tissues and cells. In addition, a ChIP-Seq experiment has shown that rs11848682 is located within a binding site of the transcription factor GATA2^23^. For rs7152362, there is little evidence suggesting that it has any regulatory function. Among all the proxy variants of rs7152362, rs12880097 represents the most promising candidate. It is in high LD with rs7152362 (r^2^ = 0.92) with a RegulomeDB score of 2b. It is located within an active enhancer in smooth muscle from the gastrointestinal track and within a transcription factor (USF1) binding site as evidenced by ChIP-Seq and footprinting experiments^23,25^. Neither rs12587586 or rs7152362, or their proxies, has been reported as an eQTL of any nearby genes^30,31^.

The second strongest association with cIMT was found with a missense SNP rs2274736 (p = 1.3 × 10^−4^ in Families, p = 0.01 in NOMAS) in the *PTPN21* gene. In addition, the effect size is in the same direction in the discovery family data set and the validation NOMAS data set (β = 0.8 and 0.01, respectively).The missense SNP has a minor allele frequency of 0.36 in Dominicans. The alternate allele at rs2274736 leads to an amino acid change from valine to alanine, but the substitution is considered as tolerated (SIFT) and benign (Polyphen). Unlike the significant CVs in *PRiMA1*, which are located within one gene with limited LD (Fig. 2a), rs2274736 is located within a region with extended LD containing multiple genes, i.e. *SPATA7*, *PTPN21*,*ZC3H14*, *EML5*, *TTC8* (Fig. 2b). Indeed, rs2274736 has been reported as an eQTL for *SPATA7* in multiple tissues including brain, adipose, pancreas and skin (GTEx Consortium. 2015).

Within the NOMAS cohort, we have data available on atherosclerotic events (ischemic stroke, myocardial infarction, and vascular death). Out of the 539 subjects with complete phenotype and covariates data, 125 subjects have had one or more atherosclerotic events. We tested rs12587586 and rs7152362 in *PRIMA1*, and rs2274736 in the *SPATA7-PTPN21-ZC3H14*-*EML5*-*TTC8* locus for association with all atherosclerotic events. This analysis revealed that the minor allele of rs7152362 is associated with fewer atherosclerotic events (OR = 0.67; p = 0.02), which is consistent with the finding that the minor allele of rs7152362 is associated with smaller cIMT (β = −0.58, p = 2.8 × 10^−4^ in Family; and β = −0.01, p = 0.05 in NOMAS).

### Rare variant analysis

**Figure 3:**
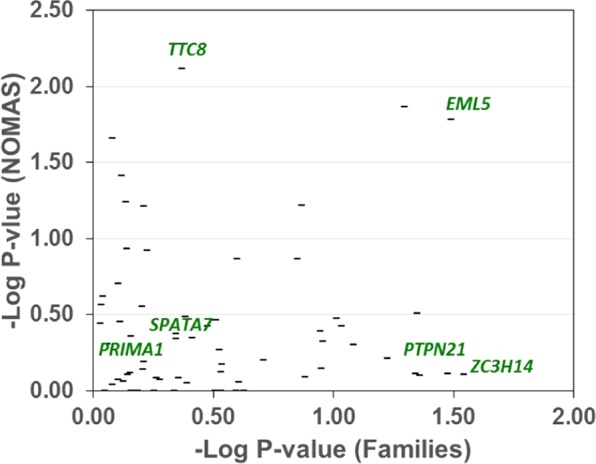
Gene-based Analysis of Exonic RVs in Families and NOMAS. Each vertical bar represents the p-values of gene-based test of exonic RVs in Families (X-axis) and NOMAS (Y-axis) datasets. All genes within the 5-gene cluster, except *SPATA7*, display evidence for association in at least one dataset. There is no evidence suggesting that exonic RVs in *PRiMA1* are collectively associated with total cIMT (p = 0.7 in both datasets).

To evaluate the association between total cIMT and RVs, gene-based tests were carried out to aggregate RVs in each gene. Figure 3 displays the overview of gene-based testing for exonic RVs in the Family and NOMAS datasets. Supplementary Table 2 displays the detailed results for top genes in the gene-based test. *EML5* and *TTC7B* were the only genes that showed evidence for association with total cIMT in both datasets (p < 0.05). Within each dataset, *ZC3H14* and *TTC8* displayed the strongest evidence for association (p = 0.029 for *ZC3H14* in families and p = 0.008 for *TTC8* in NOMAS, Fig. 3 and Supplementary Table 2).

The CV analysis identified *PRiMA1* and *SPATA7-PTPN21-ZC3H14*-*EML5*-*TTC8* as the most promising candidate loci for cIMT. To comprehensively test if RVs in the two loci collectively contribute to cIMT, we performed additional RV analyses. For *PRiMA1 (*a candidate gene identified in our previous fine-mapping study), we have sequenced the entire genomic region in the Family samples. In addition to the exonic RV analysis done for all genes (Fig. 3), we performed gene-based test including non-exonic RVs in *PRiMA1*. This analysis did not reveal any association to *PRiMA1* (data not shown). For *SPATA7-PTPN21-ZC3H14*-*EML5*-*TTC8*, exonic RVs in each gene within the 5-gene cluster displayed evidence for association (p < 0.05) in at least one dataset. To further investigate if the association was enriched in exonic RVs with functional potential, exonic RVs within the 5 genes were collectively analyzed in a locus-based analysis with stratification on functional potential (based on CADD score). In total, 80 exonic RVs were present within the 5-gene cluster in our two datasets (Supplementary Table 3). The CADD score of these exonic RVs ranged from 0 to 27.2 with a median value of 9.1 (Supplementary Table 3). Stratification based on functional potential was performed at two levels: a CADD threshold of 10 representing the top 10% of variants with functional potential genome wide, and a CADD threshold of 13 representing the top one third of variants with functional potential in our dataset. In the Family dataset, the locus-based analysis suggested that all exonic RVs were collectively associated with cIMT (p = 0.018, N = 46, Table 3). This association was enriched in RVs with a CADD score > 10 (p = 0.010, N = 15). Further stratification based on a CADD score cutoff of 13 did not provide additional enrichment for association, probably due to the small number of RVs with CADD > 13 (N = 6). In NOMAS, the locus-based analysis did not provide supporting evidence for the association when all exonic RVs were included (p = 0.280, N = 52). However, evidence for association was gradually strengthened when only exonic RVs with higher functional potentials were included (p = 0.140, N = 28 for RVs with CADD > 10; p = 0.052, N = 19 for RVs with CADD > 13, Table 3).

**Table 3:** Gene-set-based analysis of rare variants in Families and NOMAS.

	Families		NOMAS	
	P-value	Number of Variants	P-value	Number of Variants
Overall	0.018	46	0.280	52
*CADD cutoff value of 10*				
Variants with CADD > 10	0.010	15	0.140	28
Variants with CADD < 10	0.068	31	0.540	24
*CADD cutoff value of 13*				
Variants with CADD > 13	0.330	6	0.052	19
Variants with CADD < 13	0.020	40	0.570	33

Stratification based on function was performed at two levels: CADD threshold of 10 representing the top 10% of variants with functional potential genome wide, and CADD threshold of 13 representing the top one third of variants with functional potential in our dataset.

## Discussion

cIMT is a measure of subclinical atherosclerosis and a useful precursor phenotype of vascular diseases. We have previously identified a QTL for total cIMT on chr 14q near the microsatellite marker D14S606^10^. We have reported *PRiMA1*, *CCDC88C* and *FOXN3* as candidate genes after surveying the QTL with tagSNPs capturing common genetic variantss, i.e. the variants that have a minor allele frequency greater than 5%^11^. However, this tagSNP-based approach is not ideal to cover genetic variants that have a lower frequency in the population but contribute to the phenotype. To comprehensively characterize the genetic architecture underlying the QTL, we surveyed both CVs and RVs using NGS and the exome array. In addition, we assembled an independent population-based Dominican cohort to validate findings in the Dominican families. Through this strategy, we confirmed *PRiMA1* to be a strong candidate gene in the additional population-based cohort and additional independent CVs in this gene. Importantly, the minor allele of rs7152362 in *PRiMA1*, which was associated with smaller cIMT, was also associated with fewer atherosclerotic events in the NOMAS cohort, supporting the clinical relevance of the reported genetic associations with subclinical atherosclerosis phenotype. In addition to *PRiMA1*, we found evidence suggesting that exonic RVs, especially those with functional potential, in the *SPATA7*-*PTPN21-ZC3H14*-*EML5*-*TTC8* cluster collectively contribute to total cIMT.

The Framingham Heart Study Offspring cohort has found suggestive evidence for linkage to common cIMT (two point LOD = 1.75) at the same peak marker D14S606 within the QTL studied here^32^. However, we are the only group that reported evidence for association within the QTL. Others have reported that CVs at *ZHX2*, *APOC1*, *PINX*, *SLC17A4*, *BCAR1-CFDP1-TMEM170A* locus are associated with common cIMT in European cohorts^5,6^. Several groups examined RVs and cIMT with no significant association identified^8,9^. There are several differences between the current study and other studies. First, our study focused on an underrepresented population in genetic studies, i.e. Hispanics - Dominicans. Second, we took a family-based approach while most genetic studies use population-based cohorts to test for association. The family-based design allows us to take advantage of both linkage and association signals to map genetic determinants of cIMT. It is more robust to population stratification and is less confounded by gene-environment interactions. Importantly, the family-based design offers unique advantages to evaluate the contribution of RVs because RVs could be enriched among family members compared to the general population.

Among the three candidate genes nominated in the previous study, we found additional evidence supporting the association between *PRiMA1* and total cIMT. In the human genome, the most significant CV, rs12587586, resides in the 3^rd^ intron of *PRiMA1*. In the human epigenomes, rs12587586 is located close to an active enhancer in the heart tissue but with a modest regulatory potential as indicated by a RegulomeDB score of 5^23^. In searching for other SNPs that might account for the genetic association at rs12587586, rs11848682 emerged as a promising candidate. A ChiP-Seq experiment has confirmed that rs11848682 is located within a binding site of the transcription factor GATA2 in SH-SY5Y cells (RegulomeDB). GATA2 is a transcription factor that plays a crucial role in vascular development and function^33,34^. Several key genes involved in regulating vascular tone and blood pressure contain GATA2 binding sites and their transcriptional activity is modulated by GATA2. Most GATA2 targets in the vessels are expressed in the endothelium cells, including endothelin 1 (*EDN1*), a potent vasoconstrictor, endothelial-nitric oxide synthase (*NOS3*), a key enzyme in regulating vascular relaxation, and kinase Insert Domain receptor (*KDR*), a receptor for vascular endothelial growth factor (*VEGF*) and mediating VEGF-regulated endothelial proliferation, migration and permeability^35–37^. *PRiMA1* encodes the proline-rich membrane anchor for acetylcholinesterase^38^, a pivotal enzyme in acetylcholine metabolism and homeostasis. Acetylcholine causes vasodilation by promoting the release of vascular relaxing factors from the endothelium, including eNOS. It is conceivable that *PRiMA1*, like other GATA2 targets, plays a role in regulating vascular tone by modifying the activity of acetylcholinesterase and therefore acetylcholine-mediated vasoconstriction. Most studies of *PRiMA1* have been conducted in the central nervous system^38–41^. *PRiMA1* affects the activity of acetylcholinesterase via a dual-processing function, organizing acetylcholinesterase monomers into tetramers and presenting the active tetrameric form to extracellular space, without changing the mRNA level of acetylcholinesterase. In the brain of *PRiMA1* knock-out mice, acetylcholinesterase mRNA levels remain the same but the enzyme activity is diminished to less than 3% of wild type animals^42^.

Despite epigenetic evidence suggesting that rs12587586 or its proxy rs11848682 could be involved in gene expression regulation, neither of them has been established as an eQTL of any gene either by the Genotype-Tissue Expression (GTEx) project or the recent Stockholm-Tartu Atherosclerosis Reverse Networks Engineering Task (STARNET)^30,31^. It remains unclear how these CVs confer the genetic association with cIMT. One possibility is that the sequence variants at these CVs may direct allelic-specific expression of *PRiMA1* in a tissue or cell type that has not been examined in the GTEx or STARNET studies. Future studies are needed to illustrate the transcriptional regulation of *PRiMA1*, e.g., if it is a bona fide target of GATA2 in cell types other than SH-SY5Y cells, which are often used as a model of neuronal function and differentiation, and whether the associated CV directs allelic-specific expression, as well as the functional impact of *PRiMA1* on acetylcholinesterase in the arteries.

In addition to confirming the association at *PRiMA1*, both CV and RV analyses pointed to a 5-gene cluster, *SPATA7-PTPN21-ZC3H14*-*EML5*-*TTC8*. The CV analysis found strong and consistent evidence for association at a missense SNP rs2274736 (p = 1.3 × 10^−4^ in Families and p = 0.01 in NOMAS) in *PTPN21*. This missense SNP is fairly common with a minor allele frequency ranging from 0.34~0.49 in different ethnic populations. The amino acid substitution caused by the SNP is benign according to multiple prediction algorithms. Therefore, the functional significance of this SNP on the protein product of *PTPN21* is less clear. However, rs2274736 or its proxy may regulate the transcription of *SPATA7*, as it has been reported as an eQTL for this gene^31^. *SPATA7* encodes the spermatogenesis-associated protein 7. Mutations in this gene have been associated with Leber congenital amaurosis and juvenile retinitis pigmentosa^43–45^. Within the retina, SPATA7 protein predominantly localizes at the connecting cilium of photoreceptor cells and is involved in protein trafficking across the connecting cilium to the outer segments^46^. Interestingly, the RV analysis revealed that all genes within the 5-gene cluster except *SPATA7* display evidence for association in at least one dataset. The protein product of *PTPN21* (Protein Tyrosine Phosphatase, Non-Receptor Type 21) promotes cytoskeleton events that induce cell adhesion and migration^47^. *EML5* (Echinoderm Microtubule Associated Protein Like 5) encodes a protein that is likely involved in regulating microtubule dynamics^48^. *ZC3H14* (Zinc Finger CCCH-type Containing 14) encodes a zinc finger protein that regulates mRNA stability via regulating polyA length^49^. *TTC8* (Tetratricopeptide Repeat Domain 8), similar to *SPATA 7*, is involved in the formation of cilia and mutations in this gene have been implicated in nonsyndromic retinitis pigmentosa^50–54^. All five genes belong to one high-level Gene Ontology classification, i.e. non-membrane-bounded organelle. Functional exonic RVs within the five-gene cluster seem to collectively drive the genotype-phenotype association. How the orchestrated molecular functions of these five genes contribute to cIMT requires further functional studies.

We acknowledge several limitations in the current study. First, our RV analysis is largely confined within the exonic regions thus missing RVs residing outside of exons that contribute to the inter-individual variations of cIMT. A whole-genome-sequencing study is necessary to thoroughly interrogate the QTLs. Second, future functional studies are required to illustrate the mechanisms behind the detected association. Third, the current study is based only on DNA sequence variations. It has been increasingly recognized that epigenetic variation accounts for a substantial portion of phenotypic variation in populations. For example, DNA methylation has been recently implicated in arterial wall homeostasis and vascular disease development^55–58^. Future epigenetic studies are needed to fully understand the genetics of cIMT. Lastly, we focused our efforts on Caribbean Dominican subjects. As a result, our findings may not be directly generalized to other populations. However, our study provides the much-needed knowledge on the rapidly growing Hispanic population given that the majority of the genetic studies of cIMT have primarily focused on non-Hispanic white populations or Mexican Americans.

In summary, using a two-stage design with both family-based and population-based datasets, we have confirmed that common variants in *PRiMA1* are associated with cIMT. In addition, we found evidence suggesting that rare variants in *SPATA7-PTPN21-ZC3H14-EML5-TTC8* contribute to the genetic basis of cIMT. Our data provides additional insight into the molecular mechanisms of genetic susceptibility to subclinical atherosclerosis and development of overt vascular events.

## Supplementary information

Supplementary Figure

## Data Availability

The project is funded by NIH grants. Data will be made available to the public and science community according to NIH guidelines.
