# Supplementary material for: Targeted sequencing of linkage region in Dominican families implicates PRIMA1 and the SPATA7-PTPN21-ZC3H14-EML5-TTC8 locus in carotid-intima media thickness and atherosclerotic events
Source: Sci Rep. 2019 Aug 12;9:11621. doi: 10.1038/s41598-019-48186-1 (PMC6691113; doi:10.1038/s41598-019-48186-1)
Supplement: Supplementary file 1 — Supplementary Figure [file 41598_2019_48186_MOESM1_ESM.pdf]

Targeted sequencing of linkage region in Dominican families implicates *PRIMA1* and the *SPATA7-PTPN21-ZC3H14-EML5-TTC8* locus in carotid-intima media thickness and atherosclerotic events

Wang: Rare and Common Variants for Carotid IMT

Liyong Wang[1,2], PhD; Nicole Dueker[1], PhD; Ashley Beecham[1], MS; Susan H. Blanton[1,2]. PhD; Ralph L. Sacco[1,2,3,4], MD, MS; Tatjana Rundek[1,3,4], MD, PhD

1. John P Hussman Institute for Human Genomics, Miller School of Medicine, University of Miami, Miami, FL; 2. John T. McDonald Department of Human Genetics, Miller School of Medicine, University of Miami, Miami, FL; 3. Department of Neurology, Public Health Sciences, 4. Evelyn F. McKnight Brain Institute, Miller School of Medicine, University of Miami, Miami, FL

Corresponding Author: Liyong Wang, PhD, University of Miami, 1050 NW 10th Avenue, Miami, FL 33136, Tel: 305-243-2377, FAX: 305-243-2703, lwang1@miami.edu

Supplementary Materials:

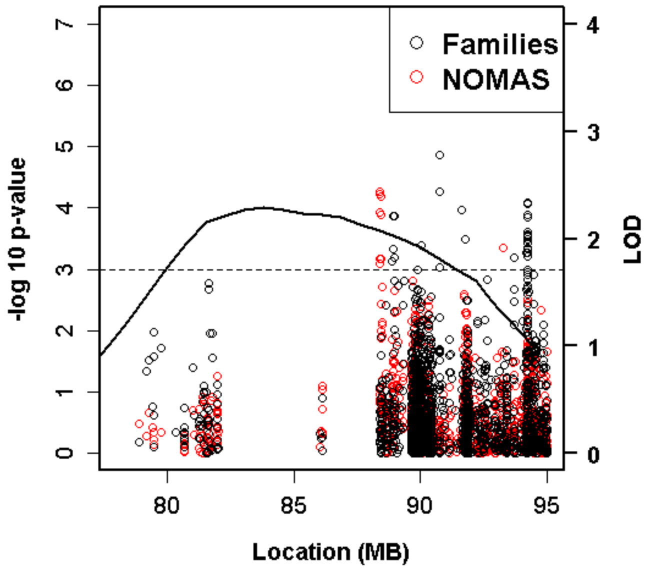

Maximum multipoint linkage and association results for total cIMT on chromosome 14. Multipoint LOD score curve over the one-LOD unit down region is depicted as a solid line. Exons of genes within the 1 LOD-unit down intervals (77.7 Mb ~ 95.0 Mb) of the chr 14 QTL, as well as introns, exons and 5 Kb flanking regions of *PRiMA1, FOXN3* and *CCDC88C* were sequenced in Families. SNVs were genotyped in NOMAS. Each symbol represents an association test on SNVs in Families (black circle) and NOMAS (red circle).

Supplementary Table 1. Top common variants in the sequencing study of Families

SNP	BP	Reference Allele	Allele Frequency	Families P-value	NOMAS P-value	Gene	Function	RegulemDB Score	CADD Score
rs2297129	88935925	A	0.6372	4.68E-04	0.031	PTPN21	coding-synonymous	5	1.87
rs2274736	88938652	A	0.6371	1.33E-04	0.031	PTPN21	missense	No data	13.26
rs879932	88945591	A	0.6393	1.33E-04	0.033	PTPN21	coding-synonymous	5	6.94
rs76026781	89830102	T	0.9458	1.30E-04	0.450	FOXN3	intron	6	1.27
rs243172	90012563	A	0.5228	4.01E-04	0.818	FOXN3	intron	6	8.52
rs45607744	90730560	T	0.925	5.42E-05	0.322	PSMC1	intron	2b	4.69
rs114316342	90736477	C	0.8947	1.36E-05	0.608	PSMC1	intron	6	9.00
rs45471798	90744546	C	0.8947	1.36E-05	0.636	NRDE2	utr-3	No data	8.85
rs3742671	90756760	A	0.8956	1.36E-05	0.672	NRDE2	coding-synonymous	6	0.90
rs2295528	91642234	A	0.61	1.09E-04	0.553	C14orf159	intron	5	2.19
rs1970911	91773494	G	0.8542	3.22E-04	0.551	CCDC88C	missense	5	9.07
rs12895096	94202390	T	0.2433	4.70E-04	0.098	PRIMA1	intron	5	15.20
rs12050435	94202579	T	0.2325	3.77E-04	0.083	PRIMA1	intron	5	1.22
rs8005268	94203917	C	0.3998	3.25E-04	0.091	PRIMA1	intron	5	1.74
rs8010323	94204438	C	0.3412	4.67E-04	0.032	PRIMA1	intron	5	0.53
rs12896080	94205962	G	0.2104	8.52E-05	0.015	PRIMA1	intron	5	0.84
rs4900194	94208530	A	0.212	8.52E-05	0.015	PRIMA1	intron	5	1.39
rs12587586	94210182	T	0.2191	8.19E-05	0.018	PRIMA1	intron	5	5.69
rs11160138	94210664	T	0.2311	1.43E-04	0.019	PRIMA1	intron	5	0.90
rs8019730	94211333	C	0.2775	3.29E-04	0.178	PRIMA1	intron	5	0.88
rs28624730	94212662	G	0.8743	4.42E-04	0.004	PRIMA1	intron	5	1.98
rs8012838	94238512	A	0.5981	1.28E-04	0.095	PRIMA1	intron	5	3.81
rs8013763	94240020	G	0.581	2.50E-04	0.069	PRIMA1	intron	5	0.52
rs59982357	94240033	C	0.5961	2.92E-04	0.038	PRIMA1	intron	5	4.14
rs7152362	94240716	C	0.5871	2.84E-04	0.047	PRIMA1	intron	5	3.62
rs12880097	94242237	G	0.5671	2.75E-04	0.101	PRIMA1	intron	2b	3.99

CVs that has a QTDT p-value less than 0.0005 in the Families. CVs that are validated in the NOMAS are in *PTPN21* and *PRIMA1*.

Supplementary Table 2. Gene-based tests in Families and NOMAS

Gene	Start Position	Families				NOMAS			
		Exonic		Non-synonymous		Exonic		Non-synonymous	
		P-value	Number of Variants	P-value	Number of Variants	P-value	Number of Variants	P-value	Number of Variants
PTPN21	88932109	**0.044**	19	0.063	3	0.800	19	0.509	11
ZC3H14	89029229	**0.029**	10			0.783	10	1.000	4
EML5	89081143	**0.032**	8			**0.017**	14	0.082	8
TTC8	89299894	0.426	4	0.546	2	**0.008**	2	**0.008**	2
FOXN3-AS2	90042645	**0.033**	2			0.776	3		
NRDE2	90744345	0.141	11	0.065	8	0.137	18	**0.041**	14
TTC7B	91006808	0.051	6			**0.014**	7	0.766	2
RPS6KA5	91337078	**0.045**	6			0.313	12	0.199	8
FBLN5	92336054	0.830	6			**0.022**	4		
RIN3	93043786	**0.046**	13	**0.029**	6	0.773	13	0.681	12
GOLGA5	93260711	0.760	7	0.463	2	**0.039**	11	**0.002**	7
UNC79	93799643	0.131	13	**0.022**	5	0.821	18	0.916	13

Genes that with a P values less than 0.05 in any gene-based tests are displayed.

Supplementary Table 3. Exonic rare variants in the 5-gene cluster in Families and NOMAS

Position	rs ID	Alleles	NOMAS MAF	Families MAF	NOMAS P-value	Families P-value	Gene	Function	Amino Acids Substitution	Protein Position	polyPhen	CADD Score	MAF in ESP
88852068	rs114796215	C/T	--	0.41%	--	1.00	SPATA7	5-prime-UTR	none	NA	unknown	7.2	unknown
88883182	rs150093878	A/T	0.53%	0.41%	0.803	1.00	SPATA7	missense	LEU,PHE	90/568	probably-damaging	14.2	AA:T=25/A=4345,EA:T=0/A=8562
88893018	rs34682727	A/G	0.09%	--	0.223	--	SPATA7	missense	ARG,GLN	240/568	benign	9.7	AA:A=23/G=4333,EA:A=1/G=8367
88895702	rs138190453	A/G	0.44%	0.41%	0.447	1.00	SPATA7	missense	CYS,TYR	276/568	benign	1	AA:A=27/G=4379,EA:A=0/G=8600
88899508	rs150364664	C/T	0.53%	--	0.232	--	SPATA7	missense	ILE,THR	339/568	probably-damaging	17.6	AA:C=1/T=4397,EA:C=4/T=8538
88904204	rs149478294	A/G	0.44%	0.41%	0.447	1.00	SPATA7	missense	HIS,ARG	381/568	benign	13.2	AA:G=27/A=4379,EA:G=0/A=8598
88904221	rs112976233	C/T	--	4.51%	--	0.101	SPATA7	synonymous	none	387/568	unknown	6.0	AA:C=210/T=4196,EA:C=1/T=8599
88904391	rs181052380	G/T	0.09%	--	0.241	--	SPATA7	missense	LEU,PHE	443/568	probably-damaging	14.3	AA:G=4406,EA:G=8600
88904456	rs142122029	A/G	0.09%	--	0.859	--	SPATA7	missense	LYS,ARG	465/568	probably-damaging	14.1	AA:G=9/A=4397,EA:G=0/A=8600
88932140	rs78630525	A/G	1.15%	0.82%	0.917	0.244	PTPN21	3-prime-UTR	none	NA	unknown	10	unknown
88932976	rs17124714	C/G	--	1.64%	--	0.574	PTPN21	3-prime-UTR	none	NA	unknown	2.4	unknown
88932980	rs769952230	A/G	--	0.41%	--	1.00	PTPN21	3-prime-UTR	none	NA	unknown	5.4	unknown
88933002	rs79018617	C/G	--	3.28%	--	0.143	PTPN21	3-prime-UTR	none	NA	unknown	9.1	unknown
88933344	rs73329641	A/G	1.15%	1.23%	0.735	1.00	PTPN21	3-prime-UTR	none	NA	unknown	7.1	unknown
88933392	rs73329643	C/G	1.15%	1.23%	0.735	1.00	PTPN21	3-prime-UTR	none	NA	unknown	4.9	unknown
88933730	rs529693811	C/T	--	0.41%	--	1.00	PTPN21	3-prime-UTR	none	NA	unknown	1.2	unknown
88934312	rs139827487	C/T	0.71%	0.41%	0.054	1.00	PTPN21	3-prime-UTR	none	NA	unknown	7.9	unknown
88934399	rs10139491	A/G	--	3.28%	--	0.097	PTPN21	3-prime-UTR	none	NA	unknown	11.5	unknown
88934443	rs111690456	C/T	0.71%	--	0.757	--	PTPN21	3-prime-UTR	none	NA	unknown	7.5	AA:T=50/C=4356,EA:T=0/C=8600
88934592	rs151002751	G/T	0.18%	--	0.753	--	PTPN21	missense	ARG,SER	1138/1175	benign	12.5	AA:G=2/T=4404,EA:G=21/T=8579
88935280	rs189968109	C/T	0.09%	--	0.734	--	PTPN21	missense	ALA,THR	1126/1175	probably-damaging	27.2	AA:T=1/C=4405,EA:T=0/C=8600
88935360	rs61746983	C/T	0.53%	0.82%	0.601	1.00	PTPN21	missense	SER,ASN	1099/1175	benign	1.7	AA:T=119/C=4287,EA:T=0/C=8600
88936027	rs116844740	C/T	1.24%	--	0.293	--	PTPN21	synonymous	ARG	1017/1175	unknown	10.3	AA:T=9/C=4397,EA:T=83/C=8517
88940063	rs111560449	C/T	--	0.41%	--	1.00	PTPN21	synonymous	none	865/1175	unknown	3.3	AA:T=34/C=4372,EA:T=0/C=8600
88940082	rs200522187	C/T	0.18%	--	0.453	--	PTPN21	missense	GLY,GLU	859/1175	probably-damaging	19.8	AA:C=4406,EA:C=8600
88945312	rs143571855	C/G	0.80%	--	0.27	--	PTPN21	missense	ASP,GLU	821/1175	benign	1.1	AA:C=78/G=4328,EA:C=0/G=8600
88945316	rs147166836	A/G	0.35%	--	0.147	--	PTPN21	missense	SER,LEU	820/1175	benign	16.1	AA:A=15/G=4391,EA:A=1/G=8599
88945407	rs3825676	C/G	--	0.41%	--	1.00	PTPN21	missense	GLY/ARG	790/1175	probably-damaging	15.4	AA:G=13/C=4393,EA:G=155/C=8445

88945477	rs376146786	A/G	--	2.46%	--	$6.3x10^{-3}$	PTPN21	synonymous	none	766/1175	unknown	3.5	AA:A=0/G=4400,EA:A=1/G=8595
88945513	rs61747078	A/G	--	0.82%	--	1.00	PTPN21	synonymous	none	754/1175	unknown	9.6	AA:A=130/G=4274,EA:A=0/G=8594
88945685	rs141118399	C/T	0.09%	--	0.508	--	PTPN21	missense	HIS,ARG	697/1175	probably-damaging	23.2	AA:C=11/T=4395,EA:C=0/T=8600
88945765	rs28380472	C/T	--	3.28%	--	0.178	PTPN21	synonymous	none	670/1175	unknown	5.6	AA:T=100/C=4302,EA:T=2/C=8590
88945886	rs751969585	A/G	--	2.46%	--	$6.3x10^{-3}$	PTPN21	missense	ALA/VAL	630/1175	probably-damaging	12.7	AA:G=4370,EA:G=8534
88945969	rs142741285	A/C	0.35%	4.10%	0.873	0.107	PTPN21	synonymous	SER	602/1175	unknown	10.1	AA:A=26/C=4370,EA:A=0/C=8580
88946074	rs769526427	G/T	--	0.48%	--	0.541	PTPN21	synonymous	none	567/1175	unknown	6.8	AA:T=4174,EA:T=8214
88946224	rs140627478	A/G	--	1.64%	--	0.245	PTPN21	synonymous	none	517/1175	unknown	10.4	AA:A=9/G=4393,EA:A=0/G=8596
88946247	rs371913962	A/C	0.09%	--	0.494	--	PTPN21	missense	CYS,GLY	510/1175	benign	5.5	AA:A=4400,EA:A=8594
88946499	rs115558453	C/T	1.24%	--	0.177	--	PTPN21	missense	VAL,ILE	426/1175	benign	4.8	AA:T=101/C=4305,EA:T=0/C=8600
88952168	rs150040997	C/T	0.53%	--	0.672	--	PTPN21	synonymous	THR	317/1175	unknown	0	AA:C=30/T=4376,EA:C=0/T=8600
88967662	rs147731756	C/G	0.18%	--	0.593	--	PTPN21	missense	ARG,THR	213/1175	possibly-damaging	19.1	AA:G=5/C=4401,EA:G=0/C=8600
88974290	rs138752198	C/T	0.09%	--	0.209	--	PTPN21	missense	GLN,ARG	142/1175	benign	4.5	AA:C=0/T=4404,EA:C=4/T=8592
89029317	rs147528258	C/G	--	2.34%	--	0.030	ZC3H14	5-prime-UTR	none	NA	unknown	11.9	unknown
89029444	rs376919923	C/T	--	2.46%	--	0.084	ZC3H14	5-prime-UTR	none	NA	unknown	9.8	AA:T=3/C=4291,EA:T=32/C=8450
89038530	rs777427072	A/T	0.09%	1.64%	0.627	0.029	ZC3H14	missense	VAL,ASP	131/736	probably-damaging	15.7	AA:T=4406,EA:T=8600
89039250	rs138503600	A/G	0.09%	--	0.632	--	ZC3H14	missense	VAL,ILE	254/736	possibly-damaging	8.4	AA:A=5/G=4401,EA:A=0/G=8600
89039308	rs116690538	A/G	0.18%	--	0.913	--	ZC3H14	missense	TYR,CYS	273/736	probably-damaging	17.4	AA:G=18/A=4388,EA:G=0/A=8600
89044465	rs201108116	G/T	0.44%	--	0.776	--	ZC3H14	missense	ASP,GLU	420/736	benign	8.4	AA:G=0/T=4406,EA:G=2/T=8596
89060883	rs577640293	A/G	0.35%	--	0.94	--	ZC3H14	5-prime-UTR	none	NA	unknown	6.8	unknown
89061118	rs145362297	A/T	--	0.41%	--	0.150	ZC3H14	synonymous	none	16/307	unknown	0.7	AA:A=30/T=4376,EA:A=2/T=8598
89061298	rs78648645	A/G	1.33%	0.82%	0.802	1.00	ZC3H14	synonymous	LYS	76/307	unknown	8.1	AA:A=22/G=4382,EA:A=215/G=8385
89068371	rs144151808	A/G	--	2.87%	--	0.396	ZC3H14	synonymous	none	486/736	unknown	9.7	AA:G=4406,EA:G=8600
89077198	rs35814324	A/G	--	4.51%	--	0.386	ZC3H14	synonymous	none	705/736	unknown	8.8	AA:G=181/A=4225,EA:G=2/A=8598
89078228	rs186697062	A/G	0.62%	--	0.822	--	ZC3H14	3-prime-UTR	none	NA	unknown	20.3	unknown
89078527	rs150274346	C/T	0.27%	--	0.739	--	ZC3H14	3-prime-UTR	none	NA	unknown	2.1	unknown
89079034	rs149251333	C/T	1.06%	0.41%	0.245	1.00	ZC3H14	3-prime-UTR	none	NA	unknown	11	unknown
89079539	rs773892136	C/G	0.09%	--	0.332	--	ZC3H14	3-prime-UTR	none	NA	unknown	12.5	unknown
89079561		A/G	--	0.82%	--	1.00	ZC3H14	3-prime-UTR	none	NA	unknown	11.2	unknown
89079562		C/T	--	0.82%	--	1.00	ZC3H14	3-prime-UTR	none	NA	unknown	6.1	unknown
89081209	rs117174690	A/G	1.06%	0.41%	0.004	1.00	EML5	3-prime-UTR	none	NA	unknown	0.7	unknown
89081598	rs145454031	C/T	0.80%	--	0.27	--	EML5	3-prime-UTR	none	NA	unknown	12	unknown

89081723	rs28434294	C/T	--	4.51%	--	0.019	EML5	3-prime-UTR	none	NA	unknown	3.0	unknown
89081729	rs76270673	C/T	1.24%	1.64%	0.696	0.574	EML5	3-prime-UTR	none	NA	unknown	7.3	unknown
89081743	rs114068875	A/G	--	0.41%	--	1.00	EML5	3-prime-UTR	none	NA	unknown	9.1	unknown
89082000	rs149196497	A/G	--	0.41%	--	1.00	EML5	3-prime-UTR	none	NA	unknown	6.2	unknown
89082116	rs201466730	C/G	0.18%	2.05%	0.004	0.057	EML5	3-prime-UTR	none	NA	unknown	19.1	AA:C=5/G=3685,EA:C=35/G=8161
89084617	rs201625686	A/G	0.27%	--	0.935	--	EML5	missense	MET,THR	1866/1978	benign	1.1	AA:G=3/A=3689,EA:G=1/A=8203
89087113	rs76231557	G/T	0.80%	--	0.023	--	EML5	missense	ALA,GLU	1787/1978	possibly-damaging	20.9	AA:T=112/G=3602,EA:T=1/G=8189
89091400	rs142374840	A/G	0.27%	--	0.739	--	EML5	synonymous	ILE	1604/1978	unknown	9.6	AA:A=2/G=4008,EA:A=0/G=8362
89093995	rs377059840	C/T	0.09%	--	0.494	--	EML5	missense	GLN,ARG	1509/1978	probably-damaging	18.1	AA:C=0/T=3708,EA:C=1/T=8197
89128111	rs201021105	G/T	0.09%	0.41%	0.005	1.00	EML5	missense	PRO,THR	1188/1978	probably-damaging	21	AA:T=2/G=3732,EA:T=4/G=8200
89129383		C/T	0.09%	--	0.494	--	EML5	missense	SER,GLY	1164/1978	benign	12.4	AA:T=3634,EA:T=7970
89151392	rs17204164	A/C	3.00%	4.10%	0.372	0.655	EML5	synonymous	VAL	983/1978	unknown	8.5	AA:A=62/C=3594,EA:A=659/C=7495
89178711	rs201948547	C/T	0.44%	--	0.734	--	EML5	missense	ILE,VAL	521/1978	benign	3.1	AA:C=16/T=3636,EA:C=0/T=8166
89206861	rs139652502	A/G	1.15%	--	0.126	--	EML5	missense	THR,MET	194/1978	benign	15.1	AA:A=11/G=3737,EA:A=112/G=8122
89212552	rs145282623	C/T	0.35%	--	0.659	--	EML5	missense	MET,VAL	145/1978	benign	2.8	AA:C=3/T=3677,EA:C=23/T=8155
89305845	rs114557412	A/G	0.09%	--	0.04	--	TTC8	missense	ASP,GLY	65/516	probably-damaging	18.1	AA:G=46/A=4360,EA:G=0/A=8600
89307227	rs150880478	A/G	0.09%	1.23%	0.056	0.404	TTC8	missense	LYS,ARG	95/516	possibly-damaging	10.8	AA:G=2/A=4404,EA:G=39/A=8561
89307242	rs139392523	A/G	--	0.82%	--	0.150	TTC8	missense	ASN/SER	100/516	possibly-damaging	0.2	AA:G=32/A=4374,EA:G=0/A=8600
89343811	rs74079414	C/T	--	7.79%	--	0.398	TTC8	3-prime-UTR	none	NA	unknown	6.2	unknown
89344226	rs59300815	A/C	--	0.41%	--	1.00	TTC8	3-prime-UTR	none	NA	unknown	7.9	unknown
